# Voltage‐gated calcium channels – from basic mechanisms to disease

**DOI:** 10.1113/JP272619

**Published:** 2016-10-13

**Authors:** Jörg Striessnig

**Affiliations:** ^1^ Department of Pharmacology and ToxicologyCentre for Molecular BiosciencesUniversity of InnsbruckInnrain 80/82, 6020InnsbruckAustria

The symposium articles in this issue of *The Journal of Physiology* provide us with novel insight into the role of voltage‐gated Ca^2+^ channels in human disease.

More than 130 years ago *The Journal of Physiology* published the milestone discovery of Sidney Ringer (Ringer, 1883) that Ca^2+^ ions are an essential constituent of extracellular fluids for normal cardiac contraction. His observations were essential for later research on electrical signalling. Fluckiger & Keynes (1955) showed that propagation of impulses along squid axons is followed by a small entry of ^45^Ca^2+^. In 1963 Shimomura and colleagues (1963) used intracellularly injected aequorin as a Ca^2+^ sensor to demonstrate an increase of free calcium in the axoplasm of a squid nerve fibre produced by sudden changes in membrane potential. At about the same time, the German physiologist Albrecht Fleckenstein coined the pharmacodynamic principle of ‘Ca^2+^ antagonism’ by showing that certain drugs could act as specific inhibitors of the slow cardiac transsarcolemmal Ca^2+^ influx without major effects on the fast Na^+^ current that initiates normal myocardial excitation (Fleckenstein, 1983). Based on these findings the existence of voltage‐gated Ca^2+^ channels (VGCCs) was postulated (Baker, 1972; Fleckenstein, 1983). The availability of different chemical classes of ‘Ca^2+^ antagonists’ (e.g. nifedipine, and verapamil analogues; widely used for antihypertensive and antianginal therapy) as the first selective Ca^2+^ channel blockers (CCBs) triggered intensive research on the molecular pharmacology of VGCCs and allowed to distinguish Ca^2+^ antagonist‐sensitive L‐type channels from other types for which selective toxins and drugs were also discovered (Zamponi *et al*. 2015 for recent review). Ten pore‐forming α1‐subunit isoforms were cloned which, together with accessory β‐ and α2δ‐subunits, give rise to 10 channel types with diverse pharmacological and biophysical properties (Table 1; Catterall *et al*. 2003 for nomenclature). This structural heterogeneity allows shaping of amplitudes, timing and kinetics of discrete Ca^2+^ signals and their delivery to subcellular signalling compartments as required for the many different physiological processes controlled by VGCCs. Moreover, the activity of each VGCC type can be further fine‐tuned by association with specific combinations of 4 β‐ and 4 α2δ‐subunit isoforms, post‐translational modifications (e.g. phosphorylation), protein–protein interactions, lipid interactions, alternative spicing and even RNA‐editing (for reviews, see Lipscombe *et al*. 2013; Striessnig *et al*. 2014). This tight regulation is highly relevant because even minor dysregulation of Ca^2+^ channel activity by upstream regulatory mechanisms or mutations in the channel subunits increases risk for various human diseases (Table 1). Early evidence for this came from missense mutations in presynaptic Cav2.1 (P/Q‐type; CACNA1A) and Cav3.2 T‐type channels (CACNA1H). Although the mutations induce only small changes in channel gating this is sufficient to cause autosomal dominant familial hemiplegic migraine (FHM1, CACNA1A mutations) or increase risk for epilepsy (CACNA1H mutations) (Table 1; Zamponi *et al*. 2015). Articles in this issue of *The Journal of Physiology* discuss how human genetic data, especially from next generation sequencing, can translate existing knowledge of the channels′ physiological functions obtained in mouse models into a better understanding of their role in human disease.

**Table 1:** Human Ca^2+^ channel diseases associated with or caused by genetic variants or mutations in pore forming α1‐subunits

		Ca^2+^ channel disease		
Type	Pore‐forming subunit (gene)	Gain of function	Loss of function	References
L‐type	Cav1.1 (CACNA1S)	Hypokalaemic periodic paralysis I (fam, mis) Malignant hyperthermia susceptibility (fam, mis)	n.r.	Striessnig *et al*. 2010
	Cav1.2 (CACNA1C)	Associated with risk for schizophrenia, bipolar disorder and ASD (SNPs) Timothy syndrome (dn, mis) Long QT‐syndrome (fam, mis)	Brugada syndrome ± short‐QT (fam, mis)	Zamponi *et al*. 2015
	Cav1.3 (CACNA1D)	Strong risk for ASD (dn, mis) PASNA (dn, mis) Primary aldosteronism (aldosterone producing adenomas, somatic, dn, mis)	Sinoatrial node dysfunction and deafness (SANDD; fam, ins)	Pinggera & Striessnig, 2016
	Cav1.4 (CACNA1F)	n.r.	Various X‐linked retinal disorders (fam, mis/lof)	Zamponi *et al*. 2015
P/Q‐type	Cav2.1 (CACNA1A)	Familial hemiplegic migraine I (fam, missense) Congential ataxia (fam, in frame deletion)	Spinocerebellar ataxia type 6 (pQex)^1^ Episodic Ataxia II (fam, mis/lof)^2^ Absence epilepsy and episodic ataxia (fam, mis) Benign paroxysmal torticollis of infancy (fam, lof) Benign paroxysmal tonic upgaze (fam/dn, lof)	Zamponi *et al*. 2015; Bahamonde *et al*. 2015; Vila‐Pueyo *et al*. 2014; Imbrici *et al*. 2004; Roubertie *et al*. 2008
N‐type	Cav2.2 (CACNA1B)	Unconfirmed: myoclonus‐dystonia syndrome (dn, mis)	n.r.	Mencacci *et al*. 2015
R‐type	Cav2.3 (CACNA1E)	n.r.	n.r.	Zamponi *et al*. 2015, 4
T‐type	Cav3.1 (CACNA1G)	Spinocerebellar ataxia (fam, mis)^3^	n.r.	Coutelier *et al*. 2015
	Cav3.2 (CACNA1H)	Associated with risk for epilepsy (dn, mis)	n.r.	Zamponi *et al*. 2015
	Cav3.3 (CACNA1I)	n.r.	n.r.	Zamponi *et al*. 2015, 4

Autoimmune disorders involving anti‐Ca^2+^ channel antibodies and diseases caused by genetic defects in associated α2δ‐ and β‐subunits are not listed here (see Zamponi *et al*. 2015 for a recent review). ^1^Functional consequences for channel function unclear but likely loss‐of‐function. ^2^Episodic ataxia may co‐exist with other phenotypes caused by CACNA1A mutations. ^3^Enhanced neuronal activity predicted in deep cerebellar nuclei. ^4^See review for mouse knockout phenotypes. Abbreviations: dn, *de novo* mutation; fam, familial (some may also occur sporadically); ins, in‐frame insertion; lof, loss of function mutation; mis, missense; n.r., not reported; PASNA, primary aldosteronism, seizures and neurological abnormalities; pQex, poly glutamine expansion.

Kabir *et al*. (2016) and Pinggera & Striessnig (2016) discuss converging evidence from genetic data indicating that the two brain L‐type channel isoforms, Cav1.2 (CACNA1C gene encoding its α1‐subunit) and Cav1.3 (CACNA1D) contribute to neuropsychiatric disease risk in humans. This is not unexpected because both channels control signalling pathways underlying activity‐dependent gene transcription, neuroplasticity, different types of learning and memory, cocaine‐induced behavioural responses and neuronal differentiation (Ma *et al*. 2013; Kabir *et al*. 2016; Striessnig *et al*. 2014). Kabir *et al*. nicely summarize genome‐wide association (GWAS) and whole exome sequencing (WES) studies strongly supporting an association of CACNA1C SNPs with neuropsychiatric disease in the general population (Table 1). GWAS studies reproducibly linked multiple frequent intronic single nucleotide polymorphisms in the CACNA1C gene to bipolar disorder, major depressive disorder, schizophrenia and autism spectrum disorders. For one of the most replicated CACNA1C risk SNPs, rs1006737, brain imaging and behavioural clinical studies convincingly revealed altered brain structure and/or function in carriers of the risk allele. This includes changes in major behavioural endophenotypes also relevant in neuropsychiatric disorders such as mood and emotions, cognition, facial processing in social interaction and reward responses with corresponding changes in the anatomy or activity of associated brain regions (Kabir *et al*. 2016). More supporting evidence for L‐type channel dysfunction in neuropsychiatric and neurological disease comes from rare *de novo* CACNA1D missense mutations causing characteristic gain of function gating changes of Cav1.3. As reviewed by Pinggera and Striessnig four different mutations were found in two patients with ASD and intellectual disability (Pinggera *et al*. 2015) and in two patients with a more severe congenital syndrome characterized by seizures, neurological abnormalities and primary aldosteronism (PASNA, see below; Scholl *et al*. 2013). Together these findings show that frequent polymorphisms in CACNA1C (the homozygous A risk allele of rs1006737 is found in about 10% of the Caucasian population) of yet less well defined consequences for Cav1.2 function contribute a significant but minor risk for developing neuropsychiatric disease in a large number of individuals, whereas *de novo* CACNA1D missense mutations absent in the healthy general population cause well defined Cav1.3 gating changes conferring a very high risk for developing ASD or PASNA in few affected individuals. As noted by Kabir *et al*. we now need to translate these new human genetic data back into mice to determine how dysfunctional L‐type Ca^2+^ channels affect signalling pathways that have already been implicated in the pathogenesis of ASD, intellectual disability and schizophrenia. These have been deduced by network analysis of human genetic data and were found to harbour genes frequently affected by damaging genetic variants in patients. Among those are pathways implicated in neuronal development, synaptic function, transcriptional regulation and chromatin remodelling (De Rubeis *et al*. 2014; Fromer *et al*. 2014; Krumm *et al*. 2014; Kirov *et al*. 2012). In this respect the finding of enhanced Cav1.3 and probably also Cav1.2 function (for rs1006737, Yoshimizu *et al*. 2014) in neuropsychiatric diseases is challenging because CCBs, safely used for decades for the treatment of hypertension and heart disease as outlined above, are available for clinical studies to test their efficacy as part of antipsychotic therapies (Ostacher *et al*. 2014; Striessnig *et al*. 2015). In contrast to earlier clinical trials with CCBs in neuropsychiatric disease these can now be strengthened by stratifying treatment groups for high risk genotypes (such as rs1006737).

As mentioned above Cav1.3 missense mutations also cause symptomatic primary aldosteronism in PASNA patients. At present we cannot explain why this has not been reported in the two ASD patients (Pinggera *et al*. 2015) although their mutations induce gating changes very similar to those in PASNA and many of the *somatic* gain of function mutations found in aldosterone‐producing adenomas (Pinggera & Striessnig, 2016). Obviously, an answer to this question also requires a much better understanding of the role of Cav1.3 signalling in aldosterone‐producing zona glomerulosa cells.

In this issue Barrett and colleagues (2016) address this question by summarizing our current understanding of the role of various VGCC types for Ca^2+^‐dependent aldosterone production in ZG cells. Interestingly, there is accumulating evidence that ZG cells have the capacity to generate electrical oscillations driven by a ‘pacemaker’ current. These oscillations critically depend on the activity of Cav3 (T‐type) channels which already activate at negative voltages (Catterall *et al*. 2003). Cav1.3 L‐type channels open later during the spikes carried by T‐type current, explaining the enhanced Ca^2+^ entry in the presence of activating CACNA1D mutations. Based on the observation that T‐type current is essential for spiking it is no surprise that germline activating CACNA1H mutations can induce familial aldosteronism (Table 1). This gene encodes the α1‐subunit of Cav3.2 T‐type channels, identifying this isoform as the physiologically relevant one in ZG cells.

In addition to genetic defects in Ca^2+^ channel α1‐subunits (or their associated subunits; see Zamponi *et al*. 2015 for review) their function may also be affected indirectly, e.g. by defective modulatory proteins. In this issue Ferron (2016) describes the recent discovery that the Fragile X mental retardation protein (FMRP) directly binds to the C‐terminal tail of Cav2.2 (N‐type) Ca^2+^ channels in neurons. It causes a tonic inhibition of Cav2.2 protein expression and its absence permits higher Cav2.2 activity. FMRP is absent in Fragile X syndrome (FXS), the most common inherited form of intellectual disability, often associated with autism. FMRP is an RNA binding protein that regulates mRNA trafficking to dendrites and protein translation in neurons (including many ion channel proteins; Brager & Johnston, 2014) and is thus essential for normal neuronal morphology, synaptic function and behaviour (Richter *et al*. 2015). Interestingly, targets for FMRP (including CACNA1D; Brager & Johnston, 2014) are enriched for *de novo* mutations in autism, intellectual disability and schizophrenia (Purcell *et al*. 2014). Animal models of FXS suggest that enhanced long‐term depression due to upregulation of mGluR5 receptor signalling pathways contributes to the neurobehavioural abnormalities (Richter *et al*. 2015). Indeed, beneficial effects of treatment with (*R*)‐baclofen, a GABA‐B receptor agonist, have been reported in exploratory clinical studies in patients with FXS and ASD (Erickson *et al*. 2014; Richter *et al*. 2015). Notably, (*R*)‐baclofen is well known to decrease glutamate release by G‐protein mediated inhibition of Cav2.2 channels. Its beneficial effects may therefore result, at least in part, from inhibition of hyperactive Cav2.2 channels in FXS. Ferron points out another exciting possibility of how Cav2.2 channels may interact with FMRP function. It is therefore possible that The Cav2.2 C‐terminus could target FMRP to sites where local activity‐dependent protein synthesis may occur, including presynaptic sites. Since FMRP activity (i.e. translational activation and repression of mRNA targets) is tightly controlled by its phosphorylation status and phosphatase activities (such as of protein phosphatase 2A) can be modulated by Ca^2+^ influx through VGCCs, this may provide a direct mechanism for coupling synaptic activity to modulation of local protein translation.

In addition to human CACNA1D gain of function mutations inherited human loss of function mutations were also described in two Pakistani families resulting in a syndrome with sinoatrial node (SAN) dysfunction and deafness (SANDD, Table 1; Baig *et al*. 2011). This phenotype is indistinguishable from the one in Cav1.3 knockout mice. An important conclusion from this report was that despite a 7‐fold higher heart rate and shorter action potential duration in the mouse as compared to humans the ion channels relevant for orchestrating normal SAN pacemaking and AV‐conduction are highly conserved among species. Based on these findings Mesirca *et al*. (2016) provide in this issue yet another example of how detailed understanding of channel function in disease may help us to devise new therapeutic strategies. They point out that the SAN dysfunction observed in Cav1.3 deficient mice replicates many features of human sick sinus syndrome (SSS) which is a frequent cardiac disorder characterized by periods of bradycardic SAN rate alternating with tachycardia due to atrial fibrillation. By confirming the role of Cav1.3 (and of other proteins) for SAN pacemaking in humans one can now go back to the corresponding mouse models and, as described above for neuropsychiatric disorders, try to rescue dysregulated signalling with drugs. Mesirca *et al*. describe such a successful effort based on years of research trying to understand the individual roles of various ion channels and their regulation by the autonomic nervous system. Cav1.3 (together with other channels including so‐called f‐channels and T‐type VGCCs) carry inward current that spontaneously depolarizes SAN cells until they reach action potential threshold. The appropriate level of net inward current (and of heart rate) can be adjusted by opposing outward currents, such as G‐protein gated acetylcholine activated K^+^‐channels (K_ACh_). SSS‐like SAN dysfunction in Cav1.3‐deficient patients or mice is explained by an imbalance due to impaired inward current. The authors discovered that inhibition of K_ACh_ activity with drugs (or by gene knockout) restores this balance and normal heart rate *in vivo* in Cav1.3 knockout mice. K_ACh_ block could therefore constitute a promising new approach to manage SSS, reducing the need for electronic pacemaker implantation.

Their data nicely show that a more detailed understanding of dysfunctional signalling networks can guide research towards new therapeutic concepts. Although on an even higher level of complexity, this also motivates further research on disease‐relevant signaling pathways in the brain to establish novel treatments for neuropsychiatric diseases.

## Additional information

### Competing interests

None declared.

### Funding

The author acknowledges research support from the Austrian Science Fund (FWF F44020, P27809, W1101) and the University of Innsbruck.
